# Which components of the Mediterranean diet are associated with dementia? A UK Biobank cohort study

**DOI:** 10.1007/s11357-022-00615-2

**Published:** 2022-07-06

**Authors:** Ivelina Dobreva, Louise Marston, Naaheed Mukadam

**Affiliations:** 1 Queen Square Institute of Neurology, Faculty of Brain Sciences, University College London, London, WC1N 3AR UK; 2 8-11 Queen Square Institute of Neurology, Dementia Research Centre, University College London, Queen Square, London, WC1N 3BG UK; 3 Department of Primary Care and Population Health, University College London, London, NW3 2PF UK; 4 Division of Psychiatry, Faculty of Brain Sciences, University College London, London, W1T 7NF UK

**Keywords:** Diet, Dementia, Mediterranean diet, Fish consumption, Cohort study

## Abstract

Cohort studies suggest that the Mediterranean diet is associated with better global cognition in older adults, slower cognitive decline and lower risk of dementia. However, little is known about the relative contribution of each component of the Mediterranean diet to dementia risk or whether the diet’s effects are due to one or more specific food components. We aimed to examine whether Mediterranean diet components are associated with all-cause dementia risk in the UK BioBank cohort. Participants joined the UK Biobank study from 2006 to 2010 and were followed until December 2020. 249,511 participants, who were at least 55 years old, without dementia at baseline were included. We used self-reported consumption of food groups, considered part of the Mediterranean diet including fruit, vegetables, processed meat, unprocessed red meat and unprocessed poultry, fish, cheese, wholegrains. Incident dementia was ascertained through electronic linkage to primary care records, hospital and mortality records or self-report. In this study with a total follow-up of 2,868,824 person-years (median 11.4), after adjusting for all covariates and other food groups, moderate fish consumption of between 2.0 and 3.9 times a week was associated with decreased risk of dementia (HR 0.84, 95%CI 0.71–0.98) compared to no consumption. Additionally, fruit consumption of between 1.0 and 1.9 servings a day was associated with reduced dementia risk (HR 0.85, 95%CI 0.74–0.99) compared to no consumption. No other Mediterranean diet components were associated with dementia risk suggesting that fish consumption may drive the beneficial effects seen from the Mediterranean diet. Further study of potential mechanisms and diet-based intervention trials are needed to establish this.

## Introduction

Dementia is a multifactorial disorder characterised by new onset and usually progressive deterioration of cognitive functions including memory, language and executive function [1]. Current figures estimate that around 57 million people worldwide live with dementia, and this figure is predicted to grow to 152 million in 2050 [2]. Targeting dietary factors may have great preventative potential for dementia. At present, dietary interventions are involved in the prevention of many conditions which increase the risk of dementia, including diabetes and cardiovascular diseases [1]. Additionally, considerable evidence suggests that those who have a healthier diet have a lower dementia risk [3, 4].

Literature on diet and dementia has expanded from examining single nutrients and their relation to dementia risk, to studying whole diet adherence. In the context of dementia prevention, the Mediterranean diet, a traditional dietary pattern followed by populations in Italy, Spain, Greece and other communities bordering the Mediterranean, has received the most attention. It is characterised by high consumption of fruit, vegetables, unrefined cereals and olive oil, high-to-moderate intake of fish, low-to-moderate intake of dairy products (mainly cheese and yogurt), low intake of meat and poultry and moderate intake of red wine, usually with meals [5]. Adherence to the Mediterranean diet has been associated with better global cognition in older adults [6], slower cognitive decline [7–9], lower risk of dementia and Alzheimer’s disease [9] and lower mortality [10]. However, methodological differences between studies such as differences in how the diet is operationalised and evaluated have resulted in inconsistent results, and other studies have not found such effects [3, 11, 12]. Examining Mediterranean diet components instead is a more flexible approach that may help in standardising research methodology and minimising heterogeneity in findings. Additionally, public health recommendations can be communicated in a more meaningful way by describing food groups to include or avoid in one’s diet [13].

The benefits of studying Mediterranean diet components are threefold:

First, breaking the diet into its core components would allow leverage for small deviations from its adherence but still capture its multifactorial aspect.

Second, it would help quantify intake of specific food groups to ensure maximum benefits, as currently there is little evidence to guide the amount and frequency of consumptions for main food components like fish, wholegrains and vegetables.

Third, the relative contribution of each food component to the diet’s beneficial effects is currently unknown.

This study aims to examine the association between Mediterranean diet components and all-cause dementia risk.

## Methods

### Study population

The UK Biobank is a multi-centre, population-based cohort of 502,538 participants aged 42–69 years at recruitment, who were recruited between 2006 and 2010. Participants attended one of the 22 centres across England, Scotland and Wales for baseline assessments for phenotyping, biological sample collection, self-reported questionnaires and nurse interviews. All participants provided informed consent using a signature-capture device. Participants also consented to the linkage of electronic health records from primary care, hospital attendances and death certification to their study data. UK Biobank received ethical approval from the National Information Governance board for Health and Social Care and the National Health Service North West Multicentre Research Ethics Committee in January 2006. We applied to and were given permission to use the UK Biobank data under study number 40055. We restricted our analysis to those individuals with data available across all variables of interest and to those 55 or older at baseline, as a dementia diagnosis in younger participants is likely to be relatively rare and due to factors unrelated to diet. Individuals who were diagnosed with dementia within 3 years of baseline assessments were also excluded.

### Exposure

#### Dietary assessment

Dietary measures were ascertained at baseline via a self-reported electronic Food Frequency Questionnaire with 29 food groups covering average consumption frequency over the past year. Diet-related items included in the current study are processed meats (such as bacon, ham, sausage, meat pies, kebabs, burgers, chicken nuggets), unprocessed poultry, unprocessed beef, unprocessed pork, unprocessed lamb/mutton, oily fish, non-oily fish, dried fruit, fresh fruit, cooked vegetables, raw vegetables, cereal, bread, cheese. Consumption of meats and fish was assessed with the question: “*How often do you eat …?*”. For each of the meat, oily and non-oily fish consumption questions, possible answers consisted of 6 categories of weekly intake: *“Never”, “Less than once a week”, “Once a week”, “2*–*4 times a week”, “5*–*6 times a week”, “Once or more daily”.* To assess intake of dried and fresh fruit, participants were asked how many pieces of dried or fresh fruit they would eat a day, and intake was recorded as an integer number of pieces of daily intake. Intake of cooked and raw vegetables was ascertained by asking for average numbers of heaped tablespoons of raw/cooked vegetables consumed per day. For both food groups, participants could select *“Less than one”* if they ate less than one piece of fruit or heaped spoon of vegetables.

To assess cereal and bread type consumption, participants were asked about which type they mainly ate. There were 5 answer categories for cereal: “Bran cereal (e.g. All Bran, Bran flakes)”, “Biscuit cereal (e.g. Weetabix)”, “Oat cereal (e.g. Ready Brek, porridge)”, “Muesli”, “Other (e.g. Cornflakes, Frosties)”. For bread consumption, possible answers consisted of 4 categories: “White”, “Brown”, “Wholemeal or wholegrain”, “Other type of bread”.

To assess intake of cheese, participants were asked *“How often do you eat cheese? (Include cheese in pizzas, quiches, cheese sauce *etc.)*”*. Possible answers consisted of 6 categories of weekly intake: *“Never”, “Less than once a week”, “Once a week”, “2*–*4 times a week”, “5*–*6 times a week”, “Once or more daily”.*

For each of the above food questions, participants were instructed to provide an estimate of average intake or select “Do not know” if they were unsure. Additionally, for each question there was an answer category of “Prefer not to answer”.

#### Quantifying food consumption

The responses on unprocessed beef, pork, poultry, lamb/mutton and processed meat were converted into weekly-based consumption frequencies of 0, 0.5, 1, 3, 5.5 and 7 times per week, using the median for each category. Unprocessed beef, pork and lamb/mutton were summed into one group of “unprocessed red meat”, and all meat groups were summed into “total meat”. To rank participants by weekly meat consumption according to the distribution of data, intake frequencies for each meat type was categorised into five groups: processed meat (0, 0.1–0.9, once, 2.0–4.9, ≥ 5.0 times/week), unprocessed red meat (0, 0.1–1.0, 1.1–1.9, 2.0–2.9, ≥ 3.0 times/week), unprocessed poultry (0, 0.1–0.9, once, 2.0–4.9, ≥ 5.0 times/week), total meat (0, 0.1–3.0, 3.1–4.9, 5.0–6.9, ≥ 7.0 times/week).

For both oily and non-oily fish, the responses were converted into weekly based consumption frequencies of 0, 0.5, 1, 3, 5.5, 7 times per week, and consumption was summed to provide total fish intake. Intake frequency for fish was categorised into 5 groups (0–0.9, once, 1.1–1.9, 2.0–3.9, ≥ 4.0 times/week).

For consumption of dried and fresh fruit, responses were converted into daily intake—two pieces of dried fruit and one piece of fresh fruit counted as one serving [14].

The items were summed to provide total fruit consumption, and daily servings of fruit were grouped into 5 categories (0–0.9, 1.0–1.9, 2.0–2.9, 3.0–4.0, ≥ 4.5 servings a day).

For consumption of cooked and raw vegetables, the same pattern was followed as for fruit. Two heaped tablespoons of cooked vegetables or raw vegetables were counted as one serving.

Weekly intake frequency of cheese was converted into groups of 6 categories of weekly intake (0, 0.1–0.9, once, 2.0–4.0, 5.0–6.0, daily or more).

Categories for each food group were determined based on data distribution to provide similar-sized groups.

Responses from questions on bread type and cereal type were combined to create an index of grain consumption. Participants’ answers on consumption of cereal type were collapsed into two categories of refined grains (muesli, cornflakes, Frosties) and wholegrains (bran cereal, biscuit cereal (Weetabix), oat cereal). Answers on consumption of bread type were also collapsed into two categories of refined grains (white bread) and wholegrains (wholegrain/wholemeal, brown bread). Combining both cereal and bread type answers, a score was generated to reflect the type of grains most commonly consumed with those scoring higher consuming wholegrains more commonly.

### Outcome

Incident all-cause dementia cases was the primary outcome, as ascertained through self-report and data linkage to hospital inpatient admissions, primary care records and death registries. Date of diagnosis was set as the earliest date of dementia codes recorded regardless of source used. Diagnoses were recorded using the International Classification of Diseases (ICD) coding system 9 and 10 [15, 16]. Across UK Biobank and all three linked databases, the positive predictive value for all-cause dementia has been found to be 82.5% [17].

### Covariates

Demographic characteristics were collected at baseline through self-reported electronic questionnaires and physical measurements. Covariates in our analyses were chosen a priori from the literature on the basis of their potential to confound the relationship between diet and dementia. They were grouped into socio-demographic (age, sex, Townsend deprivation index [18], household income, age left education), lifestyle (physical activity, smoking status, alcohol intake), mental health (loneliness and depression) and physical health factors (BMI, total cholesterol, diabetes, hypertension, cardiovascular events, major dietary changes in the last 5 years) (Table 1).

**Table 1:** List of covariate groups and their measure methods

Name	Measure method	Value
Socio-demographic		
Age at baseline	Calculated as year difference between birth date and date of first Biobank assessment centre visit	Continuous
Sex	Self-reported at first assessment visit	Categorical
Townsend deprivation index	Area based score combining information on employment, social class, housing and car availability. Assigned based on postcode (ZIP) from the preceding national census	Continuous
Household income	Self-reported annual income categorised into five bands (less than 18,000; 18,000–30,999; 31,000–51,999; 52,000–100,000; greater than 100,000)	Categorical
Age left education	Self-reported age left full-time education	Continuous
Lifestyle factors		
Physical activity	Participants reported the frequency and duration of usual engagement in each of six physical activities: walking, walking for pleasure, stair climbing, moderate physical activity, strenuous sports and vigorous physical activity. For low, moderate and high intensity activity total duration was calculated by multiplying the frequency of reported activity by its duration. The total number of minutes of each category of activity was added up, and the WHO recommended physical activity guidelines for age group 65 years and above of 150 min/week of moderate intensity activity or 75 min/week of vigorous intensity activity [24] were applied. Participants were categorised into those meeting the WHO guidelines and those not meeting them	Categorical
Smoking status	Assessed at baseline visit. Answers of “Never” and “Previous” were collapsed into a category of “No”, and “Current” was categorised as “Yes”	Categorical
Alcohol intake	Participants were asked about a range of different alcohol they drank (wine, champagne, beer, spirits), frequency and units consumed (One unit = 10 ml or 8 g of pure alcohol) [25]. Frequency of consumption was multiplied by number of units consumed in order to arrive at a total number of units of weekly intake	Continuous
Mental health factors		
Loneliness	Self-reported feeling of loneliness at baseline	Categorical
Depression	Classified as having depression if had ever seen a doctor or psychiatrist for any of the following: nerves, anxiety, tension or depression	Categorical
Physical health factors		
Body mass index	Calculated from height and weight measured during the initial Assessment Centre visit. BMI was calculated using the formula “BMI (kg/m^2^) = weight (kg) / height (m^2^)”	Continuous
Total cholesterol (mmol/l)	Measured from blood samples on first assessment visit	Continuous
Diabetes	Existing diagnosis from a clinician	Categorical
Hypertension	Measures of systolic and diastolic blood pressure were used separately. At baseline, participants’ blood pressure was measured twice. An average of the two readings was taken to calculate mean systolic and diastolic blood pressure respectively. Participants were classed as hypertensive if they reported a pre-existing diagnosis or if their baseline blood pressure was > 140/90 mmHg	Continuous
Cardiovascular events index	A cardiovascular score index was created in which participants scored one point for each cardiovascular event (stroke, heart attack or angina) they had experienced to give a total score out of 3. The higher the score, the more cardiovascular events have been experienced	Continuous
Major dietary changes in the last 5 years	Self-reported changes due to illness, other reasons or not at all	Categorical

### Statistical analysis

All analyses were carried out using Stata SE, version 15.1, and all main analyses were pre-specified. Baseline characteristics of the sample were summarised for those with and without incident dementia as mean (SD) or median (IQR) as appropriate for continuous variables and frequency and percentage for categorical variables. Follow-up time was determined as time from baseline to the earliest of dementia diagnosis, loss to follow-up or death. We used Cox proportional hazard regression models for time to dementia diagnosis.

We first conducted univariable Cox regression for each of the six food groups separately (meat, fish, vegetables, fruit, cheese, wholegrains). We then adjusted each separate food group model sequentially for sociodemographic, lifestyle, mental health and physical health covariates. Our final model included all dietary components and was fully adjusted for all covariates.

The assumption of proportional hazards was verified using Schoenfeld residuals and checking the actual versus expected Kaplan Meier plots for each diet component. We measured proportion of missing data in all variables, and analyses for all models were limited to only those who had complete data for all of the variables.

## Results

**Fig. 1:**
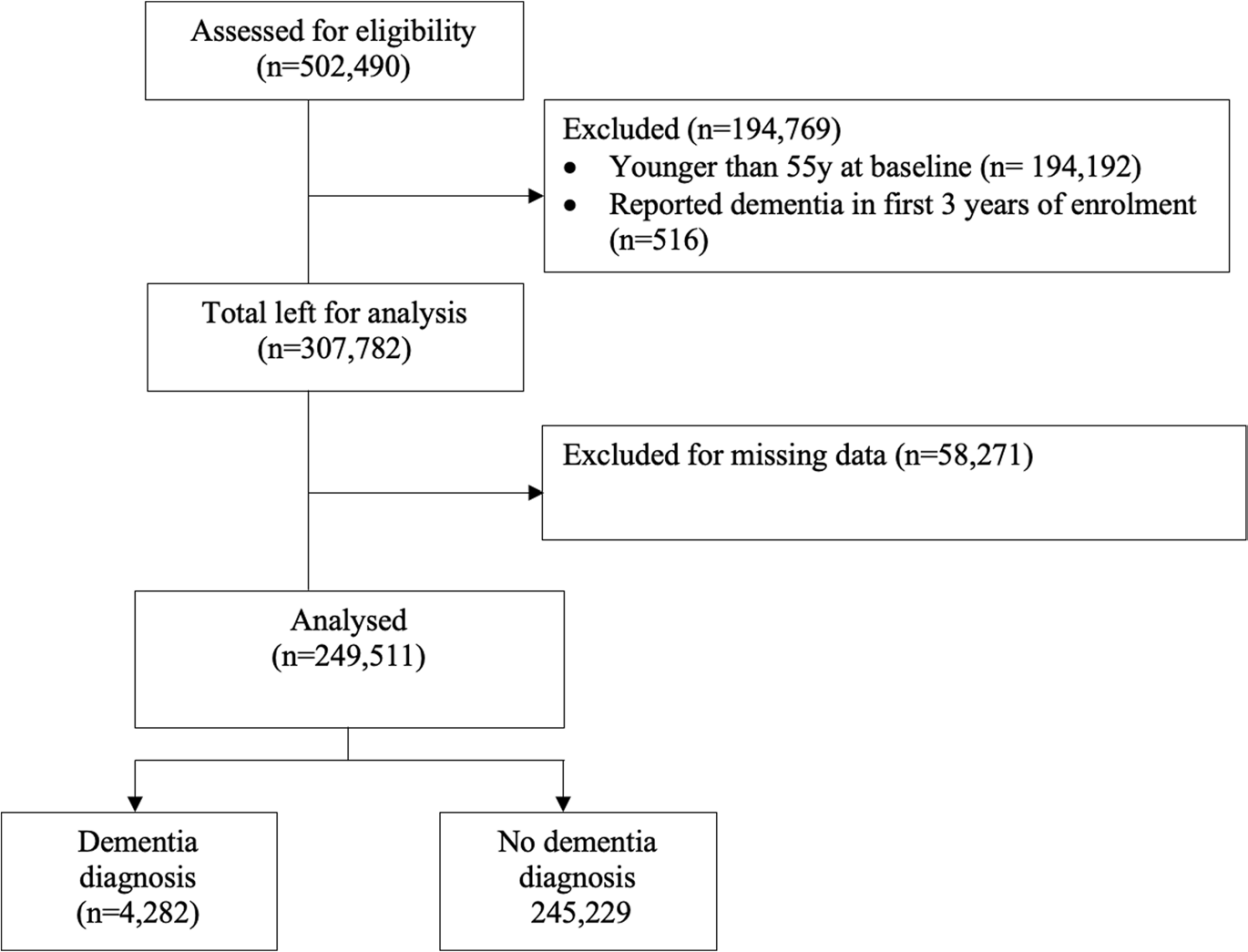
STROBE diagram showing participant selection

At baseline, 502,490 participants were assessed. After excluding participants younger than 55 years at baseline (*n* = 194,192), and those with reported dementia in the first 3 years of enrolment (*n* = 516) and with missing data (*n* = 58,271), 249,511 participants were included in the analysis (Fig. 1).

Baseline characteristics of the participants (overall and by dementia status) are shown in Table 2.

**Table 2:** Baseline characteristics of participants overall and by dementia status

	All participants (*n* = 249,511)		No dementia (*n* = 245,229)		Incident dementia (*n* = 4,282)
Age at baseline	62 ± 4		61.9 ± 4.1		64.9 ± 3.4
Gender					
Men	116,569 (46.7%)		114,294 (46.6%)		2275 (53.1%)
Women	132,942 (53.3%)		130,935 (53.4%)		2007 (46.8%)
Townsend deprivation index	− 1.6 ± 2.9		− 1.6 ± 2.9		− 1.1 ± 3.1
Household income					
Less than 18,000	58,686 (28.0%)		57,200 (27.7%)		1486 (45.3%)
18,000 to 30,999	62,938 (30.1%)		61,900 (30%)		1038 (31.6%)
31,000 to 51,999	49,664 (23.7%)		49,168 (23.8%)		496 (15.1%)
52,000 to 100,000	30,647 (14.6%)		30,432 (14.7%)		215 (6.6%)
Greater than 100,000	7522 (3.6%)		7482 (3.6%)		40 (1.2%)
Age left education	17.7 ± 3.2		17.7 ± 3.2		16.9 ± 3.5
Physical activity					
Meeting guidelines	224,292 (89.8%)		220,609 (89.9%)		3683 (86.0%)
Not meeting guidelines	25,219 (10.1%)		24,620 (10.0%)		599 (13.9%)
Smoking status					
Yes	21,466 (8.6%)		21,050 (8.5%)		416 (9.7%)
No	228,045 (91.4%)		224,179 (91.4%)		3866 (90.2%)
Alcohol intake units	12.5 ± 15		12.5 ± 15		11.6 ± 17
Loneliness					
Yes	40,890 (16.3%)		40,006 (16.3%)		884 (20.6%)
No	208, 621 (83.6%)		205,223 (83.6%)		3398 (79.4%)
Depression					
Yes	25,603 (10.2%)		24,995 (10.1%)		608 (14.2%)
No	223,908 (89.7%)		220,234 (89.8%)		3674 (85.8%)
BMI	27.5 ± 4.5		27.5 ± 4.5		27.7 ± 4.9
Total cholesterol (mmol/l)	5.7 ± 1.1		5.7 ± 1.1		5.4 ± 1.3
Diabetes					
Yes	15,623 (6.2%)		15,006 (6.1%)		617 (14.4%)
No	233,888 (93.7%)		230,223 (93.8%)		3665 (85.5%)
Hypertension					
Systolic average reading	141.9 ± 18.6		141.9 ± 18.6		144.7 ± 19.5
Diastolic average reading	82.5 ± 9.9		82.5 ± 9.9		81.7 ± 10.2
Cardiovascular score					
0 cardiovascular events	230,129 (92.2%)		226,599 (92.4%)		3530 (82.4%)
1 cardiovascular event	15,447 (6.19%)		14,898 (6.0%)		549 (12.8%)
2 cardiovascular events	3608 (1.4%)		3425 (1.4%)		183 (4.2%)
3 cardiovascular events	327 (0.1%)		307 (0.1%)		20 (0.4%)
Major dietary changes					
No	153,492 (61.5%)		151,006 (61.5%)		2486 (58%)
Yes, because of illness	29,296 (11.7%)		28,467 (11.6%)		829 (19.3%)
Yes, because of other reasons	66,723 (26.7%)		65,756 (26.8%)		967 (22.5%)
Processed meat					
0 times/week	20,883 (8.3%)		20,475 (8.3%)		408 (9.5%)
0.1–0.9 times/week	78,742 (31.5%)		77,593 (31.6%)		1149 (26.8%)
Once/week	74,432 (29.8%)		73,171 (29.8%)		1261 (29.4%)
2.0–4.9 times/week	66,553 (26.6%)		65,298 (26.6%)		1255 (29.3%)
≥ 5 times/week	8901 (3.5%)		8692 (3.5%)		209 (4.9%)
Unprocessed red meat					
0 times/week	13,690 (5.4%)		13,422 (5.4%)		268 (6.2%)
0.1–1 times/week	26,656 (10.6%)		26,085 (10.6%)		571 (13.3%)
1.1–1.9 times/week	78,764 (31.5%)		77,536 (31.6%)		1228 (28.6%)
2.0–2.9 times/week	73,173 (29.3%)		72,017 (29.3%)		1156 (27%)
≥ 3 times/week	57,228 (22.9%)		56,169 (22.9%)		1059 (24.7%)
Unprocessed poultry					
0 times/week	10,450 (4.1%)		10,257 (4.1%)		193 (4.5%)
0.1–0.9 times/week	29,688 (11.9%)		29,105 (11.8%)		583 (13.6%)
Once/week	97,477 (39.0%)		95,829 (39.0%)		1648 (38.4%)
2.0–4.9 times/week	107,891 (43.2%)		106,108 (43.2%)		1783 (41.6%)
≥ 5 times/week	4005 (1.6%)		3930 (1.6%)		75 (1.7%)
Total meat					
0 times/week	7733 (3.1%)		7615 (3.1%)		118 (2.7%)
0.1–3.0 times/week	40,765 (16.3%)		40,044 (16.3%)		721 (16.8%)
3.1–4.9 times/week	48,990 (19.6%)		48,165 (19.6%)		825 (19.2%)
5.0–6.9 times/week	84,076 (33.7%)		82,709 (33.7%)		1367 (31.9%)
≥ 7 times/week	67,947 (27.2%)		66,696 (27.2%)		1251 (29.2%)
Fish					
0–0.9 times/week	14,654 (5.8%)	14,369 (5.8%)		285 (6.6%)	
Once/ week	40,141 (16.3%)	39,501 (16.1%)		640 (14.9%)	
1.1–1.9 times/week	54,277 (21.7%)	53,465 (21.8%)		812 (18.9%)	
2.0–3.9 times/week	81,142 (32.5%)	79,844 (32.5%)		1298 (30.3%)	
≥ 4 times/week	59,297 (23.7%)	58,050 (23.6%)		1247 (29.1%)	
Fruit					
0–0.9 servings/day	17,982 (7.2%)	17,648 (7.2%)		334 (7.8%)	
1–1.9 servings/day	56,625 (22.6%)	55,751 (22.7%)		874 (20.4%)	
2–2.9 servings/day	62,202 (25.3%)	62,164 (25.3%)		1038 (24.2%)	
3–4 servings/day	69,166 (27.7%)	68,009 (27.7%)		1157 (27.0%)	
≥ 4.5 servings/day	42,536 (17.0%)	41,657 (16.9%)		879 (20.5%)	
Vegetables					
0–0.9 servings/day	12,237 (4.9%)	11,989 (4.8%)		248 (5.7%)	
1–1.9 servings/day	67,481 (27.0%)	66,390 (27%)		1091 (25.4%)	
2–2.9 servings/day	87,965 (35,2%)	86,513 (35.2%)		1452 (33.9)	
3–4 servings/day	61,146 (24.5%)	60,083 (24.5%)		1063 (24.8%)	
≥ 4.5 servings/day	20,682 (8.2%)	20,254 (8.2%)		428 (10.0%)	
Cheese					
0 times/week	7030 (2.8%)	6852 (2.7%)		178 (4.1%)	
0.1–0.9 times/week	43,546 (17.4%)	42,738 (17.4%)		808 (18.8%)	
Once/ week	54,229 (21.7%)	53,277 (21.7%)		952 (22.2%)	
2–4 times/week	113,035 (45.3%)	111,151 (45.3%)		1884 (44.0%)	
5–6 times/week	22,925 (9.1%)	22,591 (9.2%)		334 (7.8%)	
≥ 7 times/week	8746 (3.5%)	8620 (3.5%)		126 (2.9%)	
Wholegrain score	2.8 ± 1.1	2.9 ± 1.1		2.8 ± 1.3	

Continuous variables displayed as means + SDs, and categorical variables are displayed as numbers (percentages)

Over 2,868,824 person-years follow-up time at risk (median 11.4, range 0.01–14 years), 4282 (1.7%) incidences of all-cause dementia occurred in the 249,511 participants, equivalent to 1.49 cases per 1000 person-years. The earliest dementia case recorded in the sample was 3 years and 3 days after baseline assessment.

Mean age of participants was 62 (SD 4) at baseline. The sample consisted of 116,569 (46.7%) men. Generally, people who developed dementia were older, lived in more deprived neighbourhoods, had lower household income, were less physically active, had higher blood pressure, were more likely to report diagnoses of diabetes and cardiovascular events and were more likely to report smoking, and feelings of loneliness and depression. More men than women were diagnosed with dementia in the study population. Those who developed dementia were also more likely to report major dietary changes in the past 5 years due to illness.

### Individual food components and dementia risk

In the unadjusted model, compared to no consumption of processed meat, consumption of processed meat of more than 5 times a week was associated with increased risk of dementia (Table 3) (hazard ratio (HR) 1.24; 95%CI 1.05–1.46). However, when the model was adjusted for sociodemographic factors, the effect was no longer present. In the fully adjusted model, compared to no consumption, consumption of processed meat of less than once or once a week was found to be protective (0.1–0.9 times/week HR = 0.80; CI 0.70–0.92; once/week HR 0.83; 95%CI 0.72–0.95).

**Table 3:** Association between Mediterranean diet components and all-cause dementia in five adjustment models

Food components	Model 0		Model 1		Model 2		Model 3		Model 4	
	HR (95% CI)	*p* value	HR (95% CI)	*p* value	HR (95% CI)	*p* value	HR (95% CI)	*p* value	HR (95% CI)	*p* value
Processed meat										
0 times/week	Ref		Ref		Ref		Ref		Ref	
0.1–0.9 times/week	0.74 (0.66–0.83)	< .001	0.79 (0.69–0.91)	.001	0.79 (0.69–0.90)	.001	0.80 (0.70–0.91)	.001	0.80 (0.70–0.92)	.002
Once/week	0.86 (0.77–0.97)	.014	0.82 (0.72–0.94)	.005	0.81 (0.71–0.93)	.004	0.83 (0.72–0.95)	.007	0.83 (0.72–0.95)	.007
2.0–4.9 times/week	0.97 (0.87–1.09)	.717	0.88 (0.77–1.01)	.073	0.87 (0.76–1.00)	.054	0.88 (0.77–1.01)	.077	0.88 (0.77–1.01)	.088
> 5 times/week	1.24 (1.05–1.46)	.011	1.09 (0.90–1.33)	.354	1.07 (0.88–1.31)	.452	1.07 (0.88–1.31)	.447	1.07 (0.88–1.30)	.489
Unprocessed red meat										
0 times/week	Ref		Ref		Ref		Ref		Ref	
0.1–1 times/week	1.09 (0.94–1.26)	.238	0.97 (0.81–1.15)	.757	0.96 (0.80–1.13)	.641	0.96 (0.81–1.14)	.696	0.94 (0.79–1.12)	.551
1.1–1.9 times/week	0.79 (0.69–0.90)	.001	0.76 (0.65–0.89)	.001	0.75 (0.64–0.88)	.001	0.76 (0.65–0.89)	.001	0.77 (0.66–0.90)	.001
2.0–2.9 times/week	0.80 (0.70–0.91)	.001	0.77 (0.66–0.90)	.001	0.76 (0.65–0.89)	.001	0.77 (0.66–0.91)	.002	0.77 (0.66–0.90)	.002
> 3 times/week	0.94 (0.82–1.07)	.387	0.83 (0.71–0.98)	.027	0.82 (0.70–0.96)	.018	0.83 (0.71–0.98)	.031	0.82 (0.70–0.97)	.023
Unprocessed poultry										
0 times/week	Ref		Ref		Ref		Ref		Ref	
0.1–0.9 times/week	1.06 (0.90–1.25)	.458	0.90 (0.74–1.08)	.277	0.89 (0.73–1.07)	.232	0.89 (0.74–1.08)	.260	0.90 (0.74–1.08)	.277
Once/week	0.90 (0.78–1.05)	.214	0.85 (0.72–1.02)	.089	0.85 (0.71–1.02)	.083	0.87 (0.73–1.03)	.125	0.87 (0.73–1.04)	.134
2.0–4.9 times/week	0.88 (0.76–1.02)	.116	0.88 (0.74–1.04)	.154	0.88 (0.74–1.05)	.163	0.89 (0.75–1.06)	.213	0.88 (0.74–1.05)	.180
> 5 times/week	1.01 (0.77–1.32)	.917	0.95 (0.69–1.31)	.786	0.95 (0.69–1.31)	.787	0.95 (0.69–1.31)	.779	0.92 (0.67–1.26)	.630
Total meat										
0 times/week	Ref		Ref		Ref		Ref		Ref	
0.1–3.0 times/week	1.15 (0.95–1.40)	.144	0.97 (0.77–1.23)	.847	0.96 (0.76–1.21)	.745	0.96 (0.76–1.21)	.771	0.96 (0.76–1.21)	.763
3.1–4.9 times/week	1.09 (0.90–1.32)	.357	0.93 (0.74–1.17)	.569	0.92 (0.73–1.15)	.483	0.93 (0.74–1.17)	.558	0.92 (0.73–1.16)	.514
5–6.9 times/week	1.06 (0.87–1.28)	.536	0.89 (0.71–1.11)	.312	0.87 (0.70–1.10)	.263	0.88 (0.71–1.11)	.309	0.88 (0.70–1.10)	.280
> 7 times/week	1.21 (1.00–1.46)	.047	0.98 (0.78–1.23)	.890	0.97 (0.77–1.21)	.797	0.97 (0.78–1.22)	.853	0.96 (0.76–1.21)	.762
Fish										
0–0.9 times/week	Ref		Ref		Ref		Ref		Ref	
Once/week	0.81 (0.70–0.93)	.004	0.83 (0.70–0.97)	.025	0.83 (0.71–0.98)	.032	0.84 (0.71–0.99)	.039	0.86 (0.73–1.01)	.069
1.1–1.9 times/week	0.75 (0.66–0.86)	< .001	0.76 (0.65–0.89)	.001	0.78 (0.66–0.91)	.002	0.79 (0.67–0.92)	.004	0.81 (0.69–0.94)	.009
2.0–3.9 times/week	0.81 (0.71–0.92)	.002	0.76 (0.65–0.88)	< .001	0.78 (0.67–0.91)	.002	0.79 (0.68–0.92)	.003	0.80 (0.69–0.93)	.004
≥ 4 times/week	1.07 (0.94–1.21)	.290	0.94 (0.81–1.10)	.470	0.98 (0.84–1.14)	.802	0.99 (0.85–1.15)	.926	0.98 (0.85–1.15)	.897
Fruit										
0–0.9 servings/day	Ref		Ref		Ref		Ref		Ref	
1–1.9 servings/day	0.81 (0.72–0.92)	.002	0.79 (0.69–0.91)	.002	0.82 (0.71–0.95)	.010	0.83 (072–0.96)	.014	0.83 (0.72–0.96)	.014
2–2.9 servings/day	0.86 (0.76–0.97)	.020	0.89 (0.77–1.02)	.113	0.94 (0.81–1.08)	.394	0.95 (0.82–1.09)	.485	0.94 (0.81–1.08)	.394
3–4 servings/day	0.87 (0.77–0.98)	.030	0.90 (0.78–1.03)	.156	0.95 (0.83–1.10)	.567	0.96 (0.84–1.11)	.655	0.94 (0.82–1.08)	.432
≥ 4.5 servings/day	1.07 (0.95–1.22)	.233	1.07 (0.93–1.24)	.305	1.15 (0.99–1.33)	.059	1.15 (0.99 0 1.34)	.054	1.12 (0.96–1.30)	.125
Vegetables										
0–0.9 servings/day	Ref		Ref		Ref		Ref		Ref	
1–1.9 servings/day	0.78 (0.68–0.90)	.001	0.89 (0.67–0.92)	.003	0.82 (0.70–0.96)	.014	0.84 (0.72–0.98)	.036	0.84 (0.72–0.99)	.040
2–2.9 servings/day	0.80 (0.70–0.91)	.001	0.81 (0.70–0.95)	.010	0.86 (0.73–1.00)	.058	0.88 (0.76–1.03)	.135	0.89 (0.76–1.04)	.147
3–4 servings/day	0.84 (0.73–0.97)	.020	0.85 (0.73–1.00)	.055	0.90 (0.77–1.06)	.240	0.93 (0.80–1.09)	.429	0.94 (0.80–1.10)	.472
≥ 4.5 servings/day	1.01 (0.87–1.19)	.809	1.02 (0.86–1.22)	.756	1.09 (0.91–1.30)	.318	1.12 (0.93–1.34)	.208	1.12 (0.94–1.34)	.199
Cheese										
0 times/week	Ref		Ref		Ref		Ref		Ref	
0.1–0.9 times/week	0.72 (0.61–0.85)	< .001	0.84 (0.69–1.03)	.099	0.85 (0.70–1.03)	.105	0.85 (0.70–1.03)	.103	0.85 (0.70–1.04)	.126
Once/week	0.68 (0.58–0.80)	< .001	0.77 (0.64–0.94)	.010	0.78 (0.64–0.94)	.013	0.79 (0.65–0.95)	.017	0.81 (0.67–0.98)	.035
2–4 times/week	0.65 (0.55–0.76)	< .001	0.82 (0.68–0.98)	.035	0.83 (0.69–0.99)	.048	0.83 (0.69–1.00)	.059	0.88 (0.73–1.05)	.174
5–6 times/week	0.57 (0.47–0.68)	< .001	0.81 (0.65–1.00)	.059	0.82 (0.66–1.02)	.084	0.82 (0.66–1.02)	.084	0.88 (0.71–1.09)	.254
≥ 7 times/week	0.56 (0.45–0.71)	< .001	0.82 (0.63–1.06)	.135	0.83 (0.64–1.07)	.165	0.82 (0.63–1.06)	.146	0.87 (0.67–1.13)	.318
Wholegrain score	0.96 (0.94–0.99)	.012	0.99 (0.96–1.02)	.553	0.99)0.96–1.03)	.973	0.99 (0.96–1.03)	.961	0.99 (0.96–1.02)	.535

HR (95% CI) for the associations between Mediterranean diet components and incident all-cause dementia in UK Biobank (*n* = 249,511). Model 0 is unadjusted model. Model 1 is adjusted for sociodemographic factors (age, sex, Townsend deprivation score, age left education, household income)

Model 2 is adjusted for sociodemographic factors and lifestyle factors (physical activity, smoking status, weekly alcohol units). Model 3 is adjusted for sociodemographic, lifestyle factors and mental health (loneliness, depression). Model 4 is adjusted for sociodemographic, lifestyle, mental health factors and physical health factors (BMI, cholesterol, diabetes, hypertension, cardiovascular events, major dietary changes)

Compared to no consumption, moderate to high consumption of unprocessed red meat was associated with decreased risk of dementia, with the effect present throughout the subsequently adjusted models (fully adjusted model 1.1–1.9 times/week HR 0.77; 95%CI 0.66–0.90; 2.0–2.9 times/week HR 0.77; 95%CI 0.66–0.90; ≥ 3 times/week HR 0.80; 95%CI 0.70–0.97).

No associations between consumption of unprocessed poultry and dementia risk were found. In the unadjusted model, compared to no consumption, consumption of total meat of more than 7 times a week was found to increase dementia risk (HR 1.21; 95%CI 1.00–1.46); however, the association was no longer present once covariates were added. No associations between total meat consumption and dementia were found in the fully adjusted models.

Compared to consumption of less than once a week, intake of fish of between once a week and up to 2.0–3.9 times a week was associated with decreased risk of dementia—an effect present throughout the subsequent adjusted models (fully adjusted model 1.1–1.9 times/week HR 0.81, 95%CI 0.69–0.94; 2.0–3.9 times/week HR 0.80, 95%CI 0.69–0.93).

One serving a day of fruit was associated with decreased dementia risk in the fully adjusted model (HR 0.83; 95%CI 0.72–0.96) compared to consumption of less than one serving.

Consumption of one serving a day of vegetables was associated with decreased dementia risk (HR 0.84; 95%CI 0.72–0.99) compared to consumption of less than one serving.

In the fully adjusted model, consumption of cheese of once a week was found to decrease dementia risk (HR 0.81; 95%CI 0.67–0.98) compared to no consumption.

No association between wholegrain score and dementia risk was present.

### Whole model (final model)

We included all food groups and all covariates in one model to examine the effects on dementia risk. The results followed the same pattern as in the single model analyses (Table 4). Specifically, compared to consumption of less than once a week, moderate consumption of fish was found to reduce the risk of dementia (2.0–3.9 times/week HR 0.84, 95%CI 0.71–0.98), and consumption of fruit between 1 and 1.9 servings a day was also protective compared to eating fruit less than once a day (HR 0.85, 95%CI 0.74–0.99). We found that those consuming high intake of processed meat (more than five servings a week) were at an increased risk of dementia compared to those not consuming processed meat; however, the associations were not statistically significant (HR 1.23, 95%CI 0.99–1.53, *p* = 0.059).

**Table 4:** Dementia risk for Mediterranean diet food groups in fully adjusted model*

Food components	Sensitivity analysis	
	HR (95% CI)	*p* value
Processed meat		
0 times/week	Ref	
0.1–0.9 times/week	0.91 (0.77–1.07)	.267
Once a week	0.96 (0.82–1.13)	.695
2.0–4.9 times/week	1.03 (0.87–1.21)	.692
≥ 5 times/week	1.23 (0.99–1.53)	.059
Unprocessed red meat		
0 times/week	Ref	
0.1–1 times/week	0.98 (0.80–1.21)	.905
1.1–1.9 times/week	0.82 (0.66–1.00)	.058
2.0–2.9 times/week	0.82 (0.66–1.01)	.067
≥ 3 times/week	0.86 (0.70–1.06)	.171
Unprocessed poultry		
0 times/week	Ref	
0.1–0.9 times/week	1.11 (0.88–1.39)	.371
Once a week	1.10 (0.88–1.37)	.381
2.0–4.9 times/week	1.08 (0.86–1.35)	.470
≥ 5 times/week	1.05 (0.74–1.48)	.760
Fish		
0–0.9 times/week	Ref	
Once a week	0.90 (0.76–1.07)	.260
1.1–1.9 times/week	0.86 (0.73–1.01)	.074
2.0–3.9 times/week	0.84 (0.71–0.98)	.034
≥ 4 times/week	1.00 (0.85–1.18)	.925
Fruit		
0–0.9 servings/day	Ref	
1–1.9 servings/day	0.85 (0.74–0.99)	.040
2–2.9 servings/day	0.96 (0.83–1.11)	.619
3 – 4 servings/day	0.95 (0.82–1.10)	.567
≥ 4.5 servings/day	1.10 (0.94–1.28)	.226
Vegetables		
0–0.9 servings/day	Ref	
1–1.9 servings/day	0.88 (0.75–1.03)	.124
2–2.9 servings/day	0.91 (0.78–1.07)	.274
3 – 4 servings/day	0.94 (0.79–1.11)	.477
≥ 4.5 servings/day	1.07 (0.89–1.30)	.420
Cheese		
0 times/week	Ref	
0.1–0.9 times/week	0.90 (0.74–1.10)	.318
Once a week	0.86 (0.71–1.05)	.146
2–4 times/week	0.92 (0.76–1.11)	.423
v5–6 times/week	0.90 (0.73–1.12)	.385
≥ 7 times/week	0.88 (0.68–1.15)	.377
Wholegrain score	0.98 (0.95–1.01)	.432

^*^Model is adjusted for sociodemographic (age, sex, Townsend deprivation score, age left education, household income), lifestyle (physical activity, smoking status, weekly alcohol units), mental health factors (loneliness, depression) and physical health factors (BMI, cholesterol, diabetes, hypertension, cardiovascular events, major dietary changes)

Compared to no consumption, moderate consumption of unprocessed red meat was found to reduce risk of dementia; however, the associations were not statistically significant (1.1–1.9 times/week HR 0.82, 95% CI 0.66–1.00, *p* = 0.058; 2.0–2.9 times/week HR = 0.82, 95% CI 0.66–1.01, *p* = 0.067).

Unprocessed poultry, vegetables, cheese and wholegrain consumption was not found to be associated with dementia risk.

## Discussion

In this cohort of people aged 55 and over with long follow-up, we found that once all covariates and other food components were taken into account, individual components of the Mediterranean diet are not associated with dementia risk except for fish consumption and low consumption of fruit.

Initial univariable models showed an increased dementia risk from higher consumption of processed meat, reduced risk from higher consumption of unprocessed meat, reduced risk with increasing consumption of fruit and vegetables and cheese. Most of these effects were no longer statistically significant once sociodemographic factors were adjusted for, and none of these associations except for moderate unprocessed meat consumption, fish consumption and low fruit consumption were statistically significant once all covariates were adjusted for. In the fully adjusted model, once all covariates and other food groups were taken into account, only moderate fish consumption and low fruit consumption were associated with dementia risk.

Fish shows the strongest association in the single models as well as the whole model when food groups are mutually adjusted for. Our measure of fish consumption included both oily (sardines, salmon, anchovies, mackerel, herring) and non-oily fish (cod, tinned tuna, haddock), irrespective of type (canned, fresh, frozen) and cooking method (fried, boiled, roasted). Our findings support the general consensus on fish consumption and brain health benefits. Fish is one of the food groups most consistently associated with lower cognitive decline [19] and decreased risk of dementia [20]. Cohort studies conducted in France, the Netherlands, Scandinavia, Italy and the USA all show associations between regular fish consumption and decreased risk of incident dementia, Alzheimer’s Disease and cognitive decline [14], but mechanisms underlying this effect have not been elucidated.

We found low consumption of fruit to be associated with decreased dementia risk. Most cohort studies have examined the association between fruits in combination with vegetable consumption, and generally results show decreased risk of dementia [14]. Those who have examined fruit consumption on its own have generally failed to find any significant associations with dementia or cognitive decline [13, 14]. It is possible that previous studies have failed to detect any significant associations due to inadequate adjustment in their models or lack of statistical power. We could not examine potential reasons for this finding. It may be that those consuming lower levels of fruit are snacking less and eating diets that are generally healthier. Lower consumption of fruit may provide some benefits in terms of fibre intake but without additional sugar and calories from higher fruit consumption. It may also be that this is a spurious result, and replication in other cohorts would be needed before firm conclusions can be drawn.

Our results on meat consumption do not replicate previous findings from cohort studies [21]. We did not find any significant associations between processed meat consumption and increased dementia risk, although a trend was present which may relate to the stricter selection of covariates and the adjustment for other dietary components in our study.

In the context of the Mediterranean diet, it is possible for fish consumption to drive the beneficial effects. Alternatively, it is possible for the diet to influence dementia risk through secondary pathways such as cardiovascular risk factors and diabetes.

There are a number of strengths of our study. We had detailed information available on food components and robust adjustment for a wide range of covariates including sociodemographic, lifestyle, mental health and physical health factors. We included both a measure of socioeconomic deprivation and a measure of income as consumption of certain foods is likely to be associated with personal wealth. Additionally, deprivation scores relate to the area a person lives in and are not necessarily a reflection of the individual’s level of deprivation. We had a large sample size and reasonably long follow-up. Additionally, we excluded people who had dementia at baseline or within 3 years of the baseline assessment to exclude the possibility of reverse causality as much as possible. Limitations were that food consumption was only measured once in all individuals so we could not see how changes in diet may have affected dementia risk. Additionally, diet as well as many covariates were based on self-report which may be prone to bias. The UK Biobank sample is recruited from healthy volunteers so we cannot be sure the associations are generalisable.

Although we have adjusted for many covariates, we cannot rule out the possibility that the association is spurious and cannot be sure the effect is causal. This work can be further expanded to examine the influence of altered caloric intake or meal timing together with the effects of healthy diets such as the Mediterranean on brain health. This study has not collected information on calorie restriction. Emerging work in animal models has shown that prolonged reduction in daily caloric intake and periodic fasting cycles may lead to a delay of the onset and progression of disease [22]. In humans, intermittent fasting has been shown to improve cognitive dysfunction and inhibit hippocampal neuronal damage against oxidative stress [23]. Thus, future cohort studies should consider the integration of a balanced nutritious diet with periods of fasting or controlled calorie intake to further examine its effects on brain health.

Overall, our study provides evidence that consumption of fish is associated with lower risk of dementia but that other components of the Mediterranean diet are not associated with dementia risk when other covariates and other dietary components are taken into account. We cannot draw conclusions about the reasons behind this association, and this deserves further study.
